# Phenotypic plasticity of fine root growth increases plant productivity in pine seedlings

**DOI:** 10.1186/1472-6785-4-14

**Published:** 2004-09-07

**Authors:** Rongling Wu, James E Grissom, Steven E McKeand, David M O'Malley

**Affiliations:** 1 School of Life Sciences, Zhejiang Forestry University, Lin'an, Zhejiang 311300, People's Republic of China; 2 Department of Forestry, North Carolina State University, Raleigh, NC 27695, USA; 3 Institute of Food and Agricultural Sciences, University of Florida, Gainesville, FL 32611, USA

## Abstract

**Background:**

The plastic response of fine roots to a changing environment is suggested to affect the growth and form of a plant. Here we show that the plasticity of fine root growth may increase plant productivity based on an experiment using young seedlings (14-week old) of loblolly pine. We use two contrasting pine ecotypes, "mesic" and "xeric", to investigate the adaptive significance of such a plastic response.

**Results:**

The partitioning of biomass to fine roots is observed to reduce with increased nutrient availability. For the "mesic" ecotype, increased stem biomass as a consequence of more nutrients may be primarily due to reduced fine-root biomass partitioning. For the "xeric" ecotype, the favorable influence of the plasticity of fine root growth on stem growth results from increased allocation of biomass to foliage and decreased allocation to fine roots. An evolutionary genetic analysis indicates that the plasticity of fine root growth is inducible, whereas the plasticity of foliage is constitutive.

**Conclusions:**

Results promise to enhance a fundamental understanding of evolutionary changes of tree architecture under domestication and to design sound silvicultural and breeding measures for improving plant productivity.

## Background

The use of chemical fertilizers has been responsible for dramatic increase in the stem wood production of forest trees [1-4]. In an 8-year-old stand of loblolly pine growing on an infertile site in Scotland County, North Carolina, for example, stem volume increment increased 152% after the fourth year of fertilization treatment [4]. However, little is known about the mechanistic basis for such favorable effects of fertilization. One hypothesis is that improved nutrient availability leads to increases in leaf area growth and photosynthetic capacity, thus producing more photosynthate that can be allocated to the stem wood. This hypothesis has been supported by a number of physiological studies [5-7] and used as a conceptual model for plant nitrogen acquisition and cycling [8]. However, forest trees can consume as much as 60–80% of annual net primary productivity in the turnover of fine roots [3]. Fine roots are a tissue with high maintenance respiration tissue whose primary function is to absorb and metabolize water and nutrients from the soil [9-12]. A number of previous studies have shown that the production of fine roots is sensitive to the availability and distribution of nutrients within the soil [1,4,13,14]. In this study, we test a second hypothesis that the capacity of fine roots to respond to nutrient availability, referred to as *phenotypic plasticity*, can potentially increase forest-tree productivity.

Phenotypic plasticity is the potential of an organism to alter its phenotype in changing environments [15-19]. Phenotypic plasticity may play an important role in plant adaptation and evolution by combining a physiological buffering to poor environmental conditions with an improved response to favorable conditions [20]. The understanding of how phenotypic diversity is generated by the coherent change of other integrated traits is a key challenge in evolutionary biology. In much plant literature, studies of adaptive phenotypic plasticity have focused mainly on morphological and fitness traits above ground [16]. It is unclear how phenotypic plasticity exerts a significant effect on plant growth and production through the alteration of root systems below ground. Studies strongly suggest that plant root systems are adapted to different environments [14], and their diversity represents one important form of morphological evolution [12]. Fine roots are unique organs with great environmental and developmental plasticity which are subject to strong natural selection and are amenable to genetic and developmental study [10].

Loblolly pine is the most important tree species for fiber production in the southern US [21]. Because of its wide natural distribution from the moist Atlantic Coastal Plain to the dry "Lost Pines" region of Texas, this species displays strong adaptability to a range of environmental conditions. However, detailed ecophysiological and developmental mechanisms for the adaptive response of loblolly pine from a perspective of fine roots remain unknown. In the study, we integrate the conceptual theory of phenotypic plasticity into the test of the hypothesis that the reduced production of fine roots under high fertilization can increase stem productivity in loblolly pine.

## Results and Discussion

After 4 weeks of treatment, trees receiving the high nutrient treatment displayed 22% ("xeric") and 47% ("mesic") greater stem biomass than those under the low fertilizer treatment (*P *< 0.001). These values increased to 102% and 199% for these two ecotypes, respectively, when the trees were treated for 14 weeks (*P *< 0.001).

**Figure 1:**
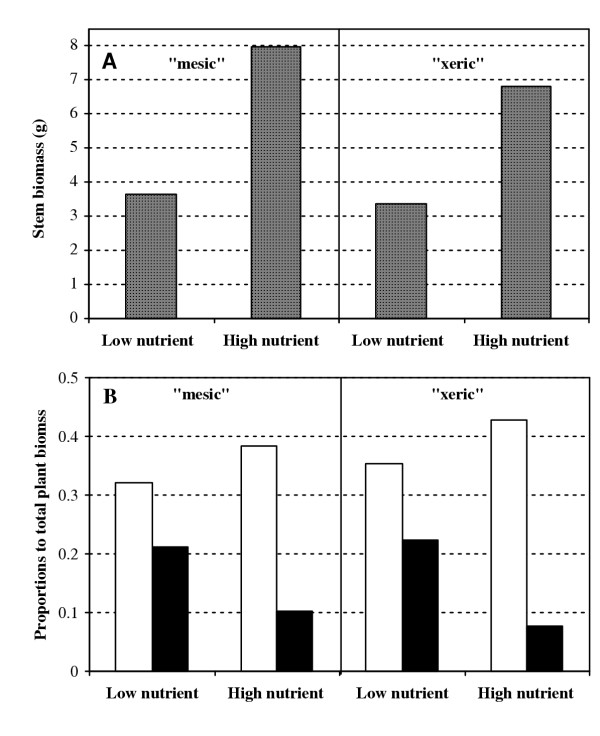
Different plant performance under the low and high nutrient treatments measured at 14 weeks of treatment. (**A**) Stem biomass. (**B**) The proportions of foliage (open bars) and fine-root (solid bars) biomass to total plant biomass.

Allometric analysis was used to evaluate the influences of foliage and fine-root biomass partitioning on stem growth which arise from differences in nutrient supply. On both harvesting dates, the proportion of foliage biomass to total plant biomass increased markedly, whereas the proportion of fine root biomass decreased significantly, with better nutrient supplies. As an illustration, we use Figure 1 to demonstrate the phenotypic plasticity of stem growth (Fig. 1A) and biomass partitioning between two treatments (Fig. 1B) on the second harvesting date. However, the degree of plasticity, defined as the absolute difference between the two treatments [16], was strikingly greater for the fine-root proportion than foliage proportion, especially for the "xeric" ecotype. The significance levels for the treatment effect on stem biomass decreased when the proportion of foliage or fine-root biomass was held constant (*P *< 0.01), as compared to the significance level when the proportion was not held constant (*P *< 0.0001). These dependent relationships suggest that increased stem biomass due to better nutrient supplies was attributable to both increased foliage investment and decreased energetic costs of fine root construction.

**Figure 2:**
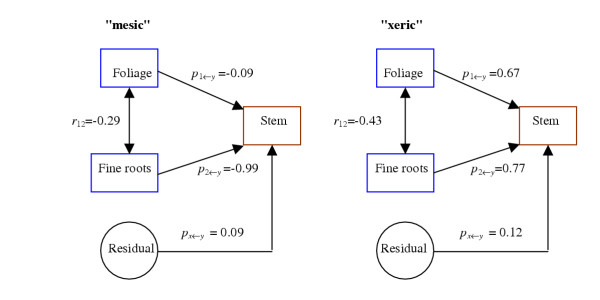
Path diagrams representing the cause-and-effect relationship between the two predictor variables, foliage biomass and fine-root biomass proportions, and the response variable, stem biomass, that results from differences in nutrient supply. The variable residual is the undetermined portion. *p *and *r *denote path coefficients and correlation coefficients, respectively.

We analyzed genetic differences in how foliage and fine-root biomass partitioning affect stem biomass through changes in nutrient level. We used correlations of family means in the two treatments to calculate path coefficients of the nutrient-induced plasticity of foliage and fine-root biomass partitioning to the plasticity of stem biomass. For "mesic" families, the plasticity of foliage biomass partitioning did not give rise to a change in stem biomass (*p*_*y*←1 _= -0.09), whereas the plasticity of fine-root biomass partitioning, *i.e*., decreased partitioning of biomass to fine roots under higher fertilization, had a significant impact on the corresponding increase of stem biomass (*p*_*y*←2 _= - 0.99, Fig. 2A). For "xeric" families, both increased biomass partitioning to foliage and decreased partitioning to fine roots as a consequence of more nutrients favorably affected stem biomass. The path coefficients derived from foliage and fine-root biomass partitioning accounted for most of the variation in stem biomass as indicated by a small residual effect (0.09–0.12), suggesting that no additional traits are required to explain stem biomass. Results from path analysis suggest that the two ecotypes have different physiological mechanisms that determine the nutrient-dependent influences of foliage and fine-root biomass partitioning on stem growth.

Foliage and fine roots have complementary roles in uptake of resources; the former in energy and carbon uptake and the latter in water and nutrient uptake [12,22]. Mechanistic modeling of resource uptake suggests that the most efficient deployment of plant biomass is to form minimal fine roots that supply water and nutrients for the production of maximum leaf area [11]. However, there are important trade-offs in generating few fine roots. We used the ratio of foliage biomass to fine-root biomass (RFF) as an architectural trait to describe the allocation of biomass within ephemeral tissues. This ratio reflects the degree to which plants display a balance of resource investment vs. resource acquisition. It was highly plastic to nutritional levels and tree development. The ratio was larger in the high nutrient treatment (RFF = 6.0–7.0) than in the low treatment (RFF = 3.0–3.5). Under the higher nutrient treatment, trees tended to invest increased energy on foliage with their growth. All these trends differed between the two ecotypes, as shown by significant interaction effects between ecotypes, treatments and harvesting dates (*P *< 0.001). Plasticity between different growth stages indicates the dependence of plastic responses on the timing and sequences of developmental events. Ecotypic variation in developmental plasticity represents different genetic bases involved in relevant developmental events [23].

Ecotypic differentiation of loblolly pine could be explained by limits of plasticity. Quantitative evolutionary genetic models predict that the phenotypic plasticity of a trait is costly or physiologically limiting when the trait is forced to respond to environmental variation ("passive" response). DeWitt et al. [24] delineated five costs (maintenance costs, production costs, information acquisition costs, developmental stability costs and genetic costs) and three limits (information reliability limits, lag-time limits and developmental range limits) of plasticity. A limit of plasticity occurs when facultative development cannot produce a trait mean as near the optimum as can fixed development. A negative relationship between the degree of plasticity and the fitness residuals (calculated from the regression of fitness on mean phenotype) identifies a limit of plasticity.

**Figure 3:**
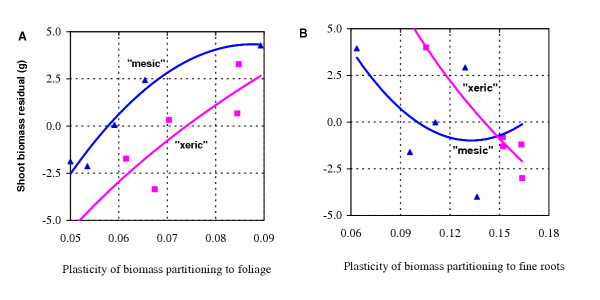
The relationships of shoot biomass residuals with the degree of the plasticity of biomass partitioning to foliage (**A**) and fine roots (**B**). In this study, shoot biomass is used as a surrogate of fitness, because great capacity of vegetative growth at early stages is advantageous for competing for growth resources and is suggested to be favored by natural selection [15]. The residuals of shoot biomass were calculated by differences between its observations and predictions estimated from foliage and fine-root biomass proportions using polynomial equations (see ref. 24 for a detailed description of this calculation approach). The degree of plasticity was represented as family difference between the nutrient treatments.

Our analysis suggests that the plasticity of fine-root biomass proportion is physiologically limiting, whereas the plasticity of foliage biomass proportion is not. The relationship of the shoot biomass residuals was positive with the degree of plasticity of foliage biomass proportion (Fig. 3A), but negative with the degree of plasticity of fine-root biomass proportion (Fig. 3B). Thus, when nutrient supply changes, foliage and fine roots will respond in different ways, with the former in a constitutive (active) way and the latter in an inducible (passive) way [24]. For both "xeric" and "mesic" ecotypes, the families that reduced fine root biomass the least had the highest stem biomass on the high nutrient treatment. Larger limits of fine-root plasticity for "xeric" than "mesic" (Fig. 3B) could explain why the stronger plasticity of fine root growth for the former ecotype did not result in more stem growth as expected (see Fig. 1A). Perhaps, for these "Lost Pines" from infertile sites, under improved nutritional conditions there is strong conflict between energetic savings due to reduced fine root production and energetic costs associated with higher efficiency of absorbing and metabolizing nutrients with fewer fine roots.

Forest tree form (biomass partitioning) is highly plastic in response to changes in nutrient levels. The carbon budgets for forest trees show a surprisingly large role of roots. Under low nutrient conditions that predominate in natural forests, 60–80% of photosynthate is allocated below ground, compared with 30% for high nutrient levels [3]. Our path analysis for loblolly pine seedlings showed that phenotypic plasticity of roots had a major influence on the plasticity of stem biomass, supporting the hypothesis that roots play a crucial role in forest productivity. Current selections when grown on high nutrient sites could have relative proportions of roots and stems and foliage that are unfavorable for high yield.

Progress towards domestication in trees will be slowed by long generation times, but is likely to be based on the exploitation of interactions between genotypes and yield associated with various types of agronomic methods (e.g., fertilizer levels), as have been shown for herbaceous crop plants. However, the environmental uncertainties during the long life span of trees have caused some breeders to consider the value of plasticity as a trait itself. And plasticity could obscure the relationship between phenotype and genotype, making selection less efficient. Efforts to domesticate forest trees will be enhanced by a deeper knowledge of phenotypic plasticity [20].

## Conclusions

Our study of biomass partitioning in relation to varying nutritional levels in loblolly pine supports the previous hypothesis, proposed by Linder and Axelson [1], that the reduced production of fine roots under fertilization results in the increase of stem production through the optimal use of energy. Yet, supporting this hypothesis does not imply that we should reject a more commonly accepted hypothesis that greater plant production due to fertilization stems from increased foliage and photosynthetic capacity. We explained the discrepancy of these two hypotheses from an ecophysiological perspective using a well-established conceptual model of phenotypic plasticity. The pattern of biomass partitioning is under environmental control and exhibits considerable ecotypic differentiation for the best utilization of available resources. In this study, we observed that biomass partitioning in loblolly pine is also under ontogenetic control, as well documented in other species [25,26]. Although our study of fine roots was performed using young loblolly pine seedlings in controlled conditions, results promise to enhance a fundamental understanding of evolutionary changes of tree architecture under domestication and to design sound silvicultural and breeding measures for improving plant productivity.

## Methods

### Plant material

Phenotypic plasticity was evaluated for fine roots and biomass partitioning of a commercially important forest tree species, loblolly pine (*Pinus taeda *L.). We used two contrasting loblolly pine ecotypes from regions that differ in soil resource availability. One of the ecotypes, known as the "Lost Pines" of Texas, is adapted to droughty conditions and low soil fertility and is denoted by "xeric", whereas the other, Atlantic Coastal Plain, is adapted to more moderate conditions and is denoted by "mesic". Adaptive differentiation between the contrasting "xeric" and "mesic" ecotypes has been previously characterized [21].

In May 1997, the seeds from the two ecotypes of loblolly pine were germinated in vermiculite, the seedlings were transplanted to 40 cm deep by 20 cm diameter plastic pots filled with pure sand, and placed in an open site at the Horticulture Field Laboratory at North Carolina State University, Raleigh. Pine seedlings from each ecotype were assigned to two different treatments: low nutrients and high nutrients [4]. The experiment was laid out in a complete randomized design with two different nutritional treatments and with five half-sib families from each ecotype in each level (8 seedlings were included per family per ecotype in each treatment). The seedlings in the high nutrient regime were fertilized at 50 ppm N solution (Peters 15-16-17) every morning, and those in the low nutrient level at 10 ppm N every other morning. The two treatments received the same amount of water. Half of the trees were harvested after 4 weeks of treatment, whereas the other half, after 14 weeks of treatment. Plants were separated into foliage, branches, stem, tap root, coarse roots and fine roots. Fine roots are defined as those of diameter ≤ 2 mm.

### Data analysis

The differences of stem biomass between the two nutritional levels were statistically analyzed using an allometric model that characterizes allometric relationships between plant parts and wholes. The model is based on an exponential function, *y *= *ax*^*b*^, where *x *and *y *are total plant biomass and stem biomass, respectively, and *a *and *b *represent the coefficient and exponent of the allometric equation, respectively [27].

Path analysis was used to identify the cause-effect relationships in a complex system [28]. Path analysis partitions the correlation of component traits with a yield trait into two parts, direct and indirect. We performed path analysis to detect the direct and indirect effects of the plasticity of foliage biomass and fine root biomass on the plasticity of stem biomass. The path coefficients for foliage biomass (*p*_1←*y*_) and fine root biomass (*p*_2←*y*_) to stem biomass through the change of nutritional levels were estimated by solving the following regular equations:

*p*_1←*y *_+ *r*_12_*p*_2←*y *_= *r*_1*y*_

*r*_12_*p*_1←*y *_+ *p*_2←*y *_= *r*_2*y*_

where *r*_1*y *_and *r*_2*y *_are the family correlation coefficients of the plasticity of foliage biomass and fine-root biomass with the plasticity of stem biomass, respectively, and *r*_12 _is the family correlation coefficient between the plasticity of foliage biomass and fine-root biomass. Residuals were estimated to evaluate the degree of determination for the path analysis [28]. All data analyses were performed using software SAS (SAS Institute 1988).

## Authors' Contributions

RW designed the study, carried out the experiment, analyzed the data and drafted the manuscript. JEG participated in the experiment. SEM participated in the design of the study. DMO participated in the design and coordination. All authors read and approved the final manuscript.
